# Carbonic anhydrase versatility: from pH regulation to CO_2_ sensing and metabolism

**DOI:** 10.3389/fmolb.2023.1326633

**Published:** 2023-11-10

**Authors:** Claudiu T. Supuran

**Affiliations:** Neurofarba Department, Section of Pharmaceutical Sciences, University of Florence, Florence, Italy

**Keywords:** carbonic anhydrase, pH regulation, CO_2_/bicarbonate sensing, metabolism, inhibitor

## Abstract

While the carbonic anhydrase (CA, EC 4.2.1.1) superfamily of enzymes has been described primarily as involved only in pH regulation for decades, it also has many other important functions. CO_2_, bicarbonate, and protons, the physiological substrates of CA, are indeed the main buffering system in organisms belonging to all life kingdoms; however, in the last period, relevant progress has been made in the direction of elucidating the involvement of the eight genetically distinct CA families in chemical sensing, metabolism, and several other crucial physiological processes. Interference with CA activity, both by inhibiting and activating these enzymes, has thus led to novel applications for CA inhibitors and activators in the field of innovative biomedicine and environment and health. In this perspective article, I will discuss the recent advances which have allowed for a deeper understanding of the biochemistry of these versatile enzymes and various applications of their modulators of activity.

## 1 Introduction

**FIGURE 1:**
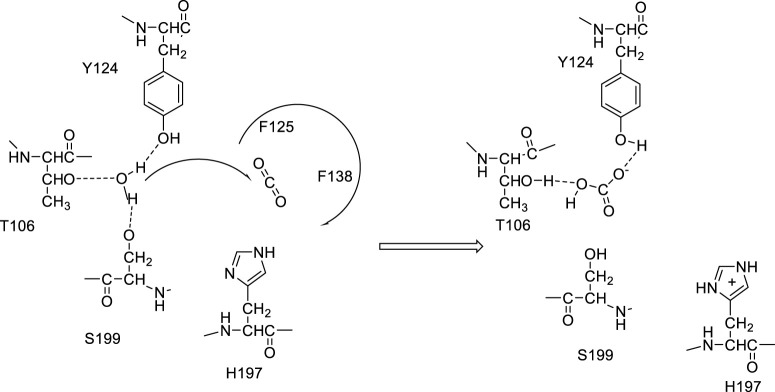
Proposed catalytic mechanism of ι-CAs: the water molecule is activated upon nucleophilic attack on the CO_2_ molecule bound in a hydrophobic pocket, through H-bond formation with T106, S199, and Y124. Proton transfer is presumably achieved by H197.

Discovered 90 years ago in the human blood (Meldrum and Roughton, 1933), during experiments aimed to understand the transport of gases in the vertebrates’ blood, the enzyme carbonic anhydrase (CA, EC 4.2.1.1) was considered for decades the quintessential metalloenzyme and a relevant tool for studying the physiology and biochemistry of the crucial molecules/ions acting as its substrates or reaction products (Krebs and Roughton, 1948; Supuran, 2023a). CA catalyzes the reversible interconversion between CO_2_ and bicarbonate, effectively hydrating this metabolic gas, generated in oxidative processes in all organisms, to soluble products: bicarbonate and protons (Lindskog, 1997; Supuran, 2008). The vertebrate enzymes, which all belong to the α-CA family, indeed use a zinc hydroxide mechanism for achieving this reaction at a physiological pH, with a huge efficacy, some of them being amongst the best catalysts known in nature, with k_cat_/K_M_ values >10^8^ M^−1^ s^−1^ and k_cat_ values >10^6^ s^−1^ (Lindskog, 1997; Supuran, 2016). During the years, novel CA genetic families have been discovered in organisms all over the phylogenetic tree, and currently, eight of them are known: α-, β-, γ-, δ-, ζ-, η-, θ-, and ι-CAs (Alber and Ferry, 1994; Cox et al., 2000; Xu et al., 2008; Del Prete et al., 2014; Capasso and Supuran, 2015; Kikutani et al., 2016; Jensen et al., 2019; Jin et al., 2020; Hirakawa et al., 2021). There are in fact very few organisms in which these enzymes were absent: few bacteria and one archaeon (Smith et al., 1999; Ueda et al., 2012). All of them, except ι-CAs, are metalloenzymes, and in addition to Zn(II), Cd(II), Fe(II), and Co(II) ions are found in their active sites, with the corresponding apo-enzymes being catalytically totally inactive (Lindskog, 1997; Ferry, 2010; Supuran, 2016) and the active-site metal hydroxide playing a crucial catalytic role (Lindskog, 1997). In fact, a water molecule coordinated at the metal ion is activated upon nucleophilic attack on the CO_2_ molecule bound in a hydrophobic pocket nearby, at the bottom of the active site cavity (Domsic et al., 2008; Aggarwal et al., 2015). This water molecule coordinated to the metal ion within the enzyme cavity has a pKa value of approximately 7, thus being orders of magnitude more nucleophilic than bulk water (Lindskog, 1997; Supuran, 2008; 2016). The rate-determining step of the entire catalytic cycle is, on the other hand, a proton transfer reaction from the metal-coordinated water molecule to the environment, which is assisted by an active-site amino acid residue with the appropriate pKa value, a His in most α-CAs (Steiner et al., 1975; Tu et al., 1989). However, Hirakawa et al. (2021) recently characterized ι-CAs from two different organisms, the cyanobacterium, *Anabaena* sp. PCC 7120, and the chlorarachniophyte alga, *Bigelowiella natans*, demonstrating that no metal ions are present in these CAs. As for the remaining seven CA genetic families, CO_2_ hydration is achieved by an active site-activated water molecule; however, as shown in Figure 1, the activation is performed without metal ions by three amino acid residues conserved in all ι-CAs investigated so far: Thr106, Ser199, and Tyr124 (Nocentini et al., 2021).

## 2 CAs and pH regulation

The CA-catalyzed reaction generates from two neutral molecules, CO_2_ and water, a weak base (bicarbonate) and a strong acid (H^+^ ions). This system is universally used for pH regulation for at least two reasons: 1) the facile and general availability of CO_2_, which as a metabolic gas is also possible to eliminate from the system rather easily, and 2) the widespread presence of CAs in most cells, tissues, and organisms and their huge catalytic activity with CO_2_ as a substrate (Supuran, 2023a). Thus, under both physiological conditions, in normal cells (Occhipinti and Boron, 2019) or in pathologic states, e.g., tumors (Neri and Supuran, 2011), CAs promote rapid buffering and the tight control/stability of pH-sensitive processes. In teleost fish, CA activity in muscle capillaries short-circuits pH regulation in red blood cells, acidifying the erythrocytes, which unloads O_2_ from hemoglobin, providing elevated tissue oxygenation, a phenomenon known as the Root effect (Rummer et al., 2013).

## 3 CO_2_/bicarbonate sensing

CO_2_ is a primary product of respiration, possibly playing other physiological roles, some of which are poorly understood, whereas its conversion to bicarbonate under the action of CAs triggers significant responses in most organisms that sense these two molecules in different ways (Cummins et al., 2014; Strowitzki et al., 2022). Altered levels of either CO_2_ or bicarbonate elicit the activation of multiple adaptive pathways both in prokaryotes and eukaryotes. Thus, in bacterial and fungal pathogens, CO_2_/bicarbonate sensing is correlated with increased virulence and/or pathogenicity (Bahn et al., 2005; Abuaita and Withey, 2009; Cottier et al., 2013). In insects, this chemosensing process is involved in prey-seeking behavior (Cummins et al., 2014), whereas in vertebrates, it is involved in taste perception (Chandrashekar et al., 2009), lung function (Kunert et al., 2022), and the control of immunity (Strowitzki et al., 2022). In algae, aquatic plants, diatoms, and cyanobacteria, CO_2_/bicarbonate sensing is highly relevant in order to supply sufficient CO_2_ for photosynthesis, and hence, sophisticated carbon-concentrating mechanisms have independently evolved in many such organisms (Momayyezi et al., 2020; Santhanagopalan et al., 2021; Zhang et al., 2022; Liao et al., 2023; Shimakawa et al., 2023).

## 4 Metabolism

In recent years, the involvement of CAs in metabolism has been considered and investigated in detail (Supuran, 2018). Several biosynthetic processes, which involve CO_2_/bicarbonate as substrates, including various carboxylation reactions and gluconeogenesis, are assisted by different CA isoforms, leading to the production of metabolic intermediates such as pyruvate, succinate, and fatty acids (Supuran, 2022). By using mitochondrial CA-selective inhibitors, it has been demonstrated that pyruvate metabolism was the most dramatically affected, followed by fatty acid metabolism and succinate metabolism (Arechederra et al., 2013). Santi et al. (2013) also showed that in tumors, bicarbonate formed from CO_2_ by hydration in the presence of CA IX or XII supplies cancer cells with intermediates utilized for sustaining their high proliferation rate through transformations in metabolic intermediates, as those described previously. The involvement of CAs in the metabolism of pathogenic bacteria, fungi, and protozoans was less investigated, but it seems to be as relevant as for mammalian cells (Supuran and Capasso, 2021; Supuran, 2023b).

## 5 Discussion and conclusion

In this section, I will discuss several applications of these enzymes and their modulation of activity in the field of biomedicine and environment and health. Its application in crop engineering, i.e., integrating CAs involved in C4 or crassulacean acid metabolism or algal carbon-concentrating mechanisms (in which various CAs are also involved) into cultivars for boosting agricultural yields has been recently demonstrated to be feasible (Findinier and Grossman AR, 2023; Förster et al., 2023) and might be of crucial relevance in a planet with >8 billion inhabitants. The field is in its infancy, but the recent breakthrough mentioned previously as well as other similar research studies (He et al., 2023) showed that both α- and β-CAs of rice (*Oryza sativa*) are essential for photosynthesis, providing the possibility for engineering plants for high-yielding crops.

In the era of global warming, CO_2_ capture has been seriously considered a possibility to relieve the long-term consequences of anthropic hot greenhouse gas emissions (Migliardini et al., 2014; Del Prete et al., 2016; Del Prete et al., 2019; Talekar et al., 2022; Huili et al., 2023; Villa et al., 2023). Various enzymes, many derived from extremophilic organisms, which provided highly thermostable CAs, have been proposed for such a purpose, either for transforming CO_2_ into bicarbonate used for algal growth, precipitating it as CaCO_3_, or transforming it into organic compounds, such as oxaloacetate (Del Prete et al., 2016).

Inhibiting human CAs has been clinically used for several decades, with many available drugs acting as diuretic, antiglaucoma, antiepileptic, antiobesity, and antitumor agents or as drugs for acute mountain sickness and idiopathic intracranial hypertension (Supuran, 2021). Other novel applications are also intensely studied and may soon lead to relevant developments for inflammation, cerebral ischemia, and cognition disturbances, among others.

## Data Availability

The original contributions presented in the study are included in the article/supplementary material; further inquiries can be directed to the corresponding author.
